# Membrane-Anchored HIV-1 N-Heptad Repeat Peptides Are Highly Potent Cell Fusion Inhibitors via an Altered Mode of Action

**DOI:** 10.1371/journal.ppat.1000509

**Published:** 2009-07-10

**Authors:** Yael Wexler-Cohen, Yechiel Shai

**Affiliations:** The Department of Biological Chemistry, The Weizmann Institute of Science, Rehovot, Israel; Harvard Medical School, United States of America

## Abstract

Peptide inhibitors derived from HIV-gp41 envelope protein play a pivotal role in deciphering the molecular mechanism of HIV-cell fusion. According to accepted models, N-heptad repeat (NHR) peptides can bind two targets in an intermediate fusion conformation, thereby inhibiting progression of the fusion process. In both cases the orientation towards the endogenous intermediate conformation should be important. To test this, we anchored NHR to the cell membrane by conjugating fatty acids with increasing lengths to the N- or C-terminus of N36, as well as to two known N36 mutants; one that cannot bind C-heptad repeat (CHR) but can bind NHR (N36 MUT*e*,*g*), and the second cannot bind to either NHR or CHR (N36 MUT*a*,*d*). Importantly, the IC_50_ increased up to 100-fold in a lipopeptide-dependent manner. However, no preferred directionality was observed for the wild type derived lipopeptides, suggesting a planar orientation of the peptides as well as the endogenous NHR region on the cell membrane. Furthermore, based on: (i) specialized analysis of the inhibition curves, (ii) the finding that N36 conjugates reside more on the target cells that occupy the receptors, and (iii) the finding that N36 MUT*e*,*g* acts as a monomer both in its soluble form and when anchored to the cell membrane, we suggest that anchoring N36 to the cell changes the inhibitory mode from a trimer which can target both the endogenous NHR and CHR regions, to mainly monomeric lipopetides that target primarily the internal NHR. Besides shedding light on the mode of action of HIV-cell fusion, the similarity between functional regions in the envelopes of other viruses suggests a new approach for developing potent HIV-1 inhibitors.

## Author Summary

Acquired immunodeficiency syndrome (AIDS) is a major global health problem, and its causative agent, human immunodeficiency virus (HIV), is extensively studied. To start an infectious cycle HIV must fuse its membrane with that of its host cell. A specific protein on the virus surface facilitates this process by undergoing major conformational changes. Several virus-cell fusion inhibitors target transiently exposed regions during the conformational changes, thereby preventing progression of the fusion process. Here, we focused on a specific fusion inhibitor peptide having two distinct binding sites and modes of inhibitions. By simple chemical modifications we demonstrate a shift between these two modes of inhibition. Most importantly, we reveal novel details regarding the conformational changes during the fusion process. Furthermore, the chemical modifications extremely enhanced the fusion inhibitory potency of the peptide. Lastly, since the fusion process of HIV shares common features with diverse biological processes, our results might contribute to their research and therapeutic efforts as well.

## Introduction

HIV-1, like other enveloped viruses, utilizes a protein embedded in its membrane, termed envelope glycoprotein ^1^(ENV), to facilitate the fusion process [1],[2],[3]. The ENV is organized as trimers on the membrane of the virus, and is composed of two non-covalently associated subunits. The surface subunit (SU), gp120, mediates host tropism [4],[5]), whereas the transmembrane subunit (TM), gp41, is responsible for the actual fusion event (reviewed in [6]). The extracellular part of gp41 is composed of several functional regions including the fusion peptide (FP), the N-terminal heptad repeat (NHR), the C-terminal heptad repeat (CHR), and the pre-transmembrane (PTM) domain.

The ability of the virus to fuse its own membrane with that of the hosting cell is due to conversion among three identified ENV conformations. Initially, the envelope subunits are in a metastable native conformation [7], in which gp41 is considered to be sequestered by gp120. Binding of gp120 to specific cell receptors involves conformational changes in both subunits, resulting in the pre-fusion conformation [7],[8] in which gp41 is exposed and extended, leading to insertion of the FP into the host cell membrane [9]. Additional conformational changes produce the post fusion conformation [10],[11], where a trimeric central coiled-coil is created by three NHR regions. These three CHR regions are packed in an anti-parallel manner into conserved hydrophobic grooves exposed on the surface of the central NHR coiled-coil. A complex representing this structure has been resolved by X-ray crystallography [12],[13], and is designated as the “six helix bundle” (SHB) or “core” structure. Similar bundles are created in intracellular vesicle fusion by SNARE proteins demonstrating a common mechanism in diverse systems [14],[15].

Inhibition of HIV-1-mediated fusion has been demonstrated by several N- or C-peptides: peptides that originate from the endogenous NHR or the CHR sequence of gp41, respectively [7],[16]. The common model is that C-peptides bind the endogenous NHR region in the pre-fusion conformation, thereby blocking core formation [17],[18],[19]. N-peptides, on the other hand, have two distinct modes of inhibitory action: binding of the endogenous CHR region in the pre-fusion conformation, thereby blocking core formation, and binding the endogenous NHR region to disrupt the creation of the internal NHR coiled-coil [20]. Previously it has been demonstrated that anchoring of inhibitory, expressed CHR peptides, to the membrane of cells can increase their inhibitory activity, as well as aid in deciphering the intermediate steps in the viruses' fusion [9],[21]. We have demonstrated earlier that conjugation of fatty acids to peptides can sufficiently anchor a short CHR-peptide to the membrane of cells, dramatically increase its inhibitory activity, and reveal the boundary of the core structure in a dynamic fusion process [22]. The observation that the increase in the inhibitory activity was significantly more pronounced when the fatty acid was attached to the C-terminus compared with the N-terminus supported a preferred orientation of the CHR peptide towards the endogenous pre-hairpin conformation. Here we address the role of the orientation of membrane bound NHR peptides during the ongoing fusion event and its implication on the understanding of the molecular mechanism of gp41 fusion.

## Materials and Methods

### Materials

Fmoc amino acids including lysine with a 4-Methyltrityl (MTT) side chain protecting group and Fmoc Rink Amide MBHA resin were purchased from Nova-biochem AG (Laufelfinger, Switzerland). Other peptide synthesis reagents, fatty acids octanoic acid (C8), dodecanoic acid (C12), and hexadecanoic acid (C16), LPC (lysophosphatidylcholine), and PBS were purchased from Sigma Chemical Co. (Israel). DiD (DiIC_18_(5) or 1,1′-dioctadecyl-3,3,3′,3′,-tetramethylindodicarbocyanine, 4-chlorobenzenesulfonate salt), DiI (1,1′- dioctadecyl-3,3,3′,3′,0 tetramethylinocarbocyanine perchlorate) lipophilic fluorescent probes and NBD-F (4-fluoro-7-nitrobenzofurazan) were obtained from Biotium (California, USA). Buffers were prepared in double-distilled water.

### Cell Lines and Reagents

Cell culture reagents and media were purchased from Biological Industries Israel (Beit Haemek LTD). All cell lines were obtained through the NIH AIDS Research and Reference Reagent Program, Division of AIDS, NIAID, NIH. Jurkat E6-1 cells were from Dr. Arthur Weiss [23], Jurkat HXBc2 (4) cells expressing HIV-1 HXBc2 Rev and ENV proteins were from Dr. Joseph Sodroski [24], TZM-bl cells were from Dr.John C. Kappes, Dr. Xiaoyun Wu, and Tranzyme Inc [25],[26], and HL2/3 cells were from Dr. Barbara K. Felber and Dr. George N. Pavlakis [27]. Cells were cultured every 3 to 4 days, and maintained in RPMI-1640 or DMEM supplemented with the appropriate antibiotics at 37°C with 5% CO_2_ in a humidified incubator. For ENV expression, Jurkat HXBc2 (4) cells were transferred to medium without tetracycline three days prior to the experiments.

### Peptide Synthesis, Fatty Acid Conjugation, and Fluorescent Labeling

GCN4 trimer, C34, and N36 were synthesized on Rink Amide 4-Methylbenzhydrylamine (MBHA) resin by using the Fmoc strategy as previously described [28]. C-terminally conjugated N36 peptides contain a lysine residue at their C-terminus with an MTT side chain protecting group, enabling the conjugation of a fatty acid that required a special deprotection step under mild acidic conditions (2×1 min. of 5% TFA (trifluoro acetic acid) in dichloro methan (DCM) and 30 min. of 1% TFA in DCM). Conjugation of a fatty acid to the N-terminus was performed using standard Fmoc chemistry. Addition of the NBD (emission-530 excitation-467) fluorescent probe to the N- or C-terminus of selected peptides was performed using 3 equivalents of NBD-F in a 2% diisopropylamin (DIEA) solution in DMF for one hour. All peptides were cleaved from the resin by a TFA: DDW: TES (93.1∶4.9∶2 (v/v)) mixture, and purified by reverse phase high performance liquid chromatography (RP-HPLC) to >95% homogeneity. The molecular weight of the peptides was confirmed by platform LCA electrospray mass spectrometry.

### Cell-Cell Fusion Inhibition Assay

The protocol utilizing Jurkat E6-1 and Jurkat HXBc2 cells for a cell-cell fusion assay was previously described [29]. In short, Jurkat E6-1 and Jurkat HXBc2 cells were labeled with DiI and DiD lipophilic fluorescent probes, respectively. The two cell populations were co-incubated, in a ratio of 1∶1, for 6 h in the presence of eight different concentrations of the inhibitory peptides. Prior to measurements the cells were washed, spinned, dissolved in PBS, and put on ice. Cells co-incubated without the presence of peptides served as an optimal fusion reference. Unlabeled cells that were handled similarly served as an intrinsic fluorescence control. Cells labeled separately with DiI or DiD were used to adjust the optimal separation of fluorescent signals. Jurkat HXBc2 cells labeled with DiI were co-incubated with Jurkat HXBc2 cells labeled with DiD for a fusion background that was subtracted from the measurements of the experiment. The following alterations were applied to the original protocol: (i) 5 µL of a 1 mg/mL DiI or DiD solution in dimethylsulfoxide (DMSO) was added to 1 mL of 4×10^6^ cells/mL Jurkat E6-1 or Jurkat HXBc2 cells, respectively. (ii) For each data point 150,000 events were collected. Measurements were performed on a FACSort machine, upgraded to a FACSCalibur cell analyzer (Becton Dickinson). Fitting of the data points was performed according to the equation derived from Hills' equation:
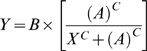
In this equation *B* is the maximum value, therefore it equals 100% fusion, *A* is the *IC_50_* value, and *c* represents Hill's coefficient, in this particular case: the inhibitory oligomeric state of the peptide. For the fitting, we uploaded the *X* and *Y* values of the raw data (after subtracting the background) into a nonlinear least squares regression (curve fitter) program that provided the *IC_50_* value (*A* of the equation), as well as the *c* value.

### Triple Staining Flow Cytometry Fusion Assay

For triple staining, the same cell-cell fusion inhibition assay experiment as above was performed in the presence of NBD-labeled peptides. Cells labeled separately with DiI or DiD, and unlabeled cells in the presence of an NBD-labeled peptide were used to compensate for the optimal separation of the three fluorescent signals. For each data point 500,000 events were collected. The eight different possible combinations (triple, NBD, DiI, DiD, NBD+DiI, NBD+DiD, DiI+DiD, no label) were defined in the analysis software and the percentage of each one was calculated. The percentage of NBD labeling (peptide) on all cell types in relation to all available labeled cells in the system was calculated. This analysis provided us with the percentage of cells labeled with NBD-peptide.
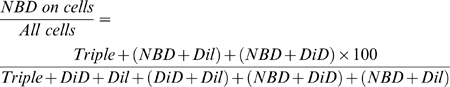



Additionally, the percentage of NBD labeling (peptide) in cells labeled with DiD (effector) or DiI (target) cells was further calculated. Analysis of the data enabled us to examine the relative binding of labeled peptides to different cell populations, namely, target or effector cells.
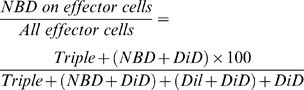


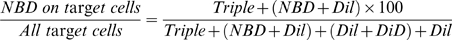



### Circular Dichroism (CD) Spectroscopy

CD measurements were performed on an Aviv 202 spectropolarimeter. The spectra were scanned using a thermostatic quartz cuvette with a path length of 1 mm. Wavelength scans were performed at 25°C, the average recording time was 15 sec., in 1 nm steps, the wavelength range was 190–260 nm. Peptides were scanned at a concentration of 10 µM in HEPES buffer (5 mM, pH 7.4) and in a membrane mimetic environment of 1% LPC in double distilled H_2_O.

## Results

### Anchoring of N36 to the Membrane Increases Its Inhibitory Activity

**Figure 1:**
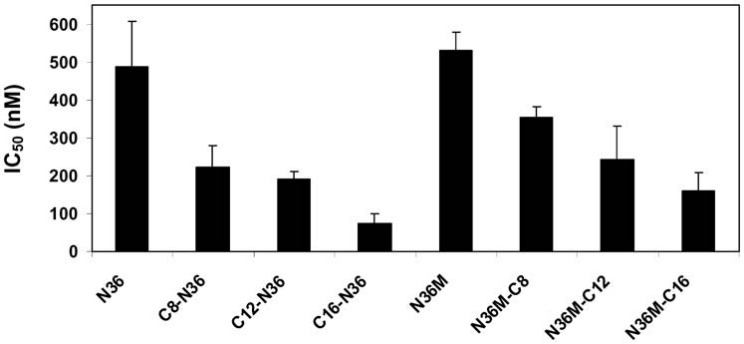
Cell-cell fusion inhibition assay for the N36 peptide and its fatty acid conjugates. Fusion inhibition (*IC_50_* values) induced by the peptides. For each peptide at least four independent experiments were performed and were included in the calculation of the standard deviation.

To scrutinize the effect of anchoring N36 to the membrane, we conjugated octanoic, dodecanoic, and palmitic acids to the N-terminus of N36 (Table 1). The resulting peptides C8-N36, C12-N36, and C16-N36 were examined in a cell-cell fusion inhibition assay and the results are shown in Figure 1. A correlation was observed between the length of the conjugated fatty acid and the inhibitory activity of the N- conjugated N36 peptides. N36, C8-N36, C12-N36, and C16-N36 exhibited *IC_50_* values of 488±119, 222±56, 190±21, and 72±27 nM, respectively. Interestingly, AcN36 was not active up to 2000 nM; therefore we refer to it as inactive. This correlates with previous studies demonstrating an *IC_50_* of 16000±2000 nM and 584±46 nM for the acetylated and non-acetylated forms of N36, respectively [20],[30]. Overall, our data reveal that the anchoring of N36 to the membrane significantly increases its inhibitory activity.

**Table 1:** Sequences, designations, and IC_50_ values of the peptides and their lipophilic conjugates.

Designation	X	Peptide sequence	Z	IC_50_(nM)
N36	H	**X**- SGIVQQQNNLLRAIEAQQHLLQLTVWGIKQLQARIL		488±119
AcN36	Acetate			>2000
C8-N36	C8-			222±56
C12-N36	C12-			190±21
C16-N36	C16-			72±27
N36M		SGIVQQQNNLLRAIEAQQHLLQLTVWGIKQLQARILK(δ-NH**Z**)		531±48
N36M-C8			-C8	354±25
N36M-C12			-C12	241±89
N36M-C16			-C16	159±47

### The Orientation of Anchored N36 toward the Endogenous CHR Region Is Not Crucial

To examine the importance of the proper orientation of the N36 peptide in relation to the pre-fusion conformation, we also conjugated octanoic, dodecanoic, and palmitic acids to the C-terminus of modified N36, termed N36M (Table 1). The parental peptide and the resulting fatty acid-conjugated peptides N36M, N36M-C8, N36M-C12, and N36M-C16 (Table 1) were examined in a cell-cell fusion inhibition assay and the results are presented in Figure 1. Likewise, a correlation was observed between the length of the conjugated fatty acid and the inhibitory activity of the C-conjugated N36 peptides. N36M, N36M-C8, N36M-C12, and N36M-C16 exhibited *IC_50_* values of 531±48, 354±25, 241±89, and 159±47 nM, respectively. Since acetylating N36 abrogates its activity we added an acetyl group to N36M-C12 and N36M-C16 resulting in AcN36M-C12 and AcN36M-C16. Both lipopeptides were examined in a cell-cell fusion inhibition assay and exhibited *IC_50_* values of 226±38, and 125±51 nM, respectively. Since these values are similar to those of N36M-C12 and N36M-C16 we can conclude that the charge in their N-terminus does not influence their inhibitory ability, contrary to N36.

Interestingly, there was only a slight difference between the activities of N- and C-terminally conjugated peptides having the same fatty acid. This was in contrast with the results obtained with the C-helix peptide, in which there was a marked difference (∼30-fold) between them [22]. Thus, we can conclude that the length of the fatty acid is important, and it is correlated to the inhibitory activity, whereas primarily, the orientation of the peptides is not critical for their activity pattern.

### Inhibitory Curves Analysis Suggest a Different Mode of Inhibition for the Peptides

**Figure 2:**
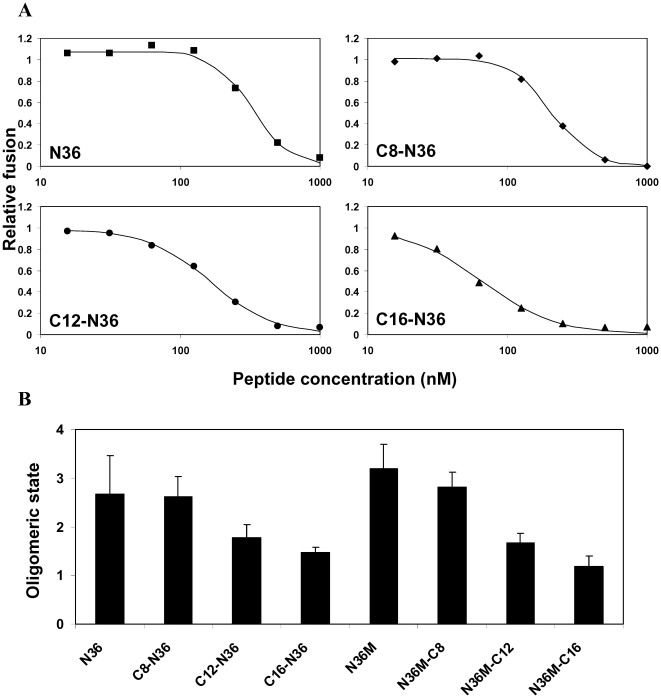
The inhibitory oligomeric state of the peptides. (A) Fusion inhibition curves of the N-terminally conjugated peptides (other data not shown). N36, C8-N36, C12-N36, and C16-N36 are represented by closed squares, diamonds, circles, and triangles, respectively, and the fitted curves are represented by continuous lines. (B) The inhibitory oligomeric state of the peptides. The Hill's coefficient parameter for the different peptides is presented. For each peptide at least four independent experiments were performed and were included in the calculation of the standard deviation.

Representative experiments showing the inhibitory activity curves of N36 and its N-terminally fatty acid-conjugated analogs is presented in Figure 2A. It reveals different shapes of the inhibition curves for the different peptides shifted from sigmoid through a median shape to hyperbolic. A sigmoid shape can be explained by the tendency of N36 to oligomerize. Therefore, we speculated that the different binding curves might be attributed to a different inhibitory oligomeric state of the peptides. Consequently, for optimal fitting, we employed an equation that contains a cooperativity parameter, indicative in this case, to the inhibitory oligomeric state of the peptide. Therefore, after a fit is achieved, the *c* value represents the oligomeric state of the peptide. The values of the oligomerization parameters (averaged from at least four independent experiments) for the different peptides are presented in Figure 2B. The *c* values for the N- conjugated N36 peptides, namely: N36, C8-N36, C12-N36, and C16-N36 are 2.67, 2.61, 1.77, and 1.47 respectively. The *c* values for the C- conjugated N36 peptides, namely: N36M, N36M-C8, N36M-C12, and N36M-C16 are 3.19, 2.82, 1.67, and 1.19 respectively. The *c* parameter for the original peptide (N36 or N36M) was compared to the *c* parameter of its longest fatty acid conjugate (C16-N36 or N36M-C16) by the nonparametric Mann-Whitney test, the two sided significance was p = 0.016, demonstrating statistical significance for these results. These data reveal an interesting shift in the oligomerization tendency. It suggests that for the native peptides, N36, and N36M, the tendency is for the trimeric form. The longer the fatty acid the lower is the oligomerization value until it almost reaches a monomer with the C16-N36, and N36M-C16 peptides.

### Relative Binding of Labeled Peptides to the Membrane of Cells

**Figure 3:**
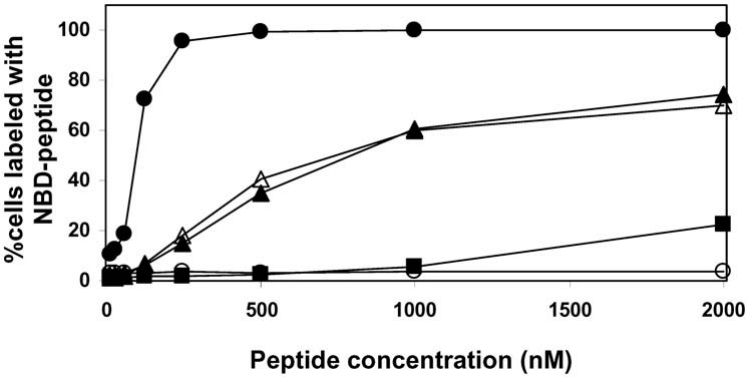
Relative binding of NBD-labeled peptides to cells. _NBD_N36, C16-N36M_NBD_, and _NBD_N36M-C16 are represented by closed squares, closed triangles, and open triangles, respectively. For comparison, a non-binding peptide _NBD_GCN4 (open circles) and the strongly binding peptide C16-_NBD_GCN4 (closed circles) were measured.

We tested whether the attachment of the fatty acids to the peptides allowed their anchoring to the cell membrane by utilizing a triple staining flow cytometry assay that incorporates fluorescently labeled target cells, effector cells, and inhibitory peptides [22]. This assay allowed the determination of the *IC_50_* of the peptides, as well as monitoring the percentage of cells labeled with the different peptides (Table 2). We analyzed the most and least active peptides, namely, _NBD_N36 (parallel in its inhibitory activity to AcN36), _NBD_N36M-C16, and C16-N36_NBD_ (Figure 3). The _NBD_GCN4 peptide served as a negative control for a non-binding peptide, whereas, C16-_NBD_GCN4 served as a positive control for a strongly binding peptide. As expected, both are not inhibitors (data not shown). The data reveal a direct correlation between the activity of the N-helix peptides and their global binding to the cells.

**Table 2:** Sequences and designations of the NBD labeled peptides and their lipophilic conjugates.

Designation	X	Peptide sequence	Z
_NBD_N36	NBD	**X**- SGIVQQQNNLLRAIEAQQHLLQLTVWGIKQLQARIL	
_NBD_N36M-C16	NBD	**X**- SGIVQQQNNLLRAIEAQQHLLQLTVWGIKQLQARILK(δ-NH**Z**)	-C16
C16-N36M_NBD_	C16-	**X**- SGIVQQQNNLLRAIEAQQHLLQLTVWGIKQLQARILK(δ-NH**Z**)	NBD
_NBD_GCN4	NBD	**X**- KQIEDKIEEILSKIYHIENEIARIKKLIGER	
C16-_NBD_GCN4	C16-	**X**- K(δ-NH**Z**)QIEDKIEEILSKIYHIENEIARIKKLIGER	NBD

### Structure of the Peptides in Solution and in a Membrane Mimetic Environment Alone and in Combination with the C-Helix C34

**Figure 4:**
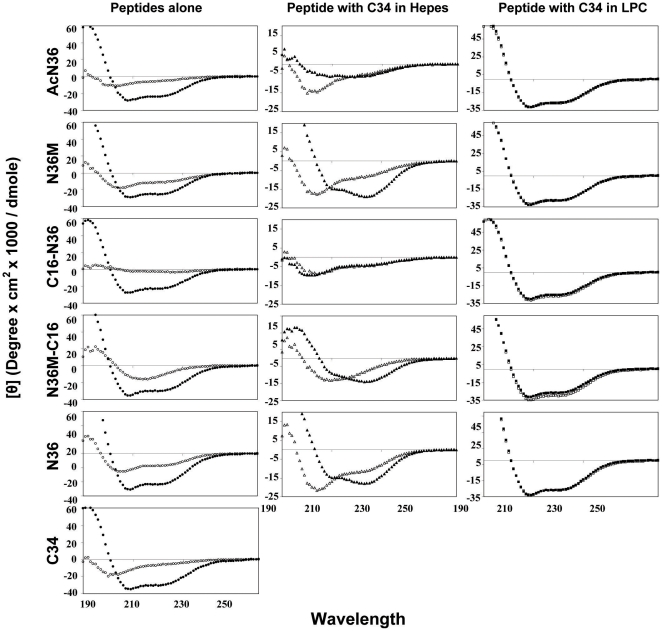
CD spectroscopy of the peptides alone, and together with C34. The peptides were measured at 10 µM in HEPES buffer (5 mM, pH 7.4) or 1% LPC in H_2_O (membrane mimetic environment). Left column: The peptide signal alone in buffer solution (open circles) compared to the peptide signal in 1% LPC (closed circles). Middle column: The calculated, non-interacting signal for the N-peptide with C34 (open triangles), compared to the observed experimental signals, obtained following incubation of the two peptides together in buffer solution (closed triangles). Right column: The same experiment was done in LPC. The calculated non-interacting and the experimental signals are represented by open and closed squares, respectively.

We determined the secondary structure of the most active and inactive peptides in solution to find out whether this feature correlates with their activity pattern. N36 and N36M exhibited α-helical structures in solution, whereas the structure of AcN36, C16-N36, and N36M-C16 was undefined (Figure 4). The peptides' ability to create a core structure with C34 in solution was also monitored. The CD signal of each peptide was measured and this signal was added to the signal of C34. This calculated combined signal would represent the signal in the case that the peptides do not interact with each other. This signal was compared to the actual signal monitored upon co-incubation of the two peptides together. If the two peptides interact one with each other, we would expect to see a difference between the two signals. AcN36, in contrary to the results presented in a previous study [31], and C16-N36 were unable to create a core structure, whereas N36 and N36M-C16 did interact with C34 (Figure 4) [32],[33]. Note that Chan et al have used the C-34 and the N-36 peptides both in their acetylated forms and obtained a stable core. Here we obtained a stable core with both peptides in their non-acetylated forms, but we could not get a stable core with one acetylated and one non-acetylated peptides, the reason for which is not clear. The structure of the peptides alone, and their ability to create a core structure with C34 was also measured in a membrane mimetic environment (Figure 4). Under these conditions, all the peptides exhibited α-helical structures. However, with all peptides, the non-interactive signal overlapped the experimental signal. In this case, the overlap does not necessarily mean that there is no creation of the core structure. Since all peptides have strong helical signals by themselves they could create a core structure without an observed change in the secondary structure. Overall, these data demonstrate that the structure of the peptides and their ability or inability to create a core structure with C34 (in solution or in a membrane mimetic environment) cannot account for their activity pattern.

### Utilizing Known N36 Mutants to Explore the Inhibitory Mechanism

**Figure 5:**
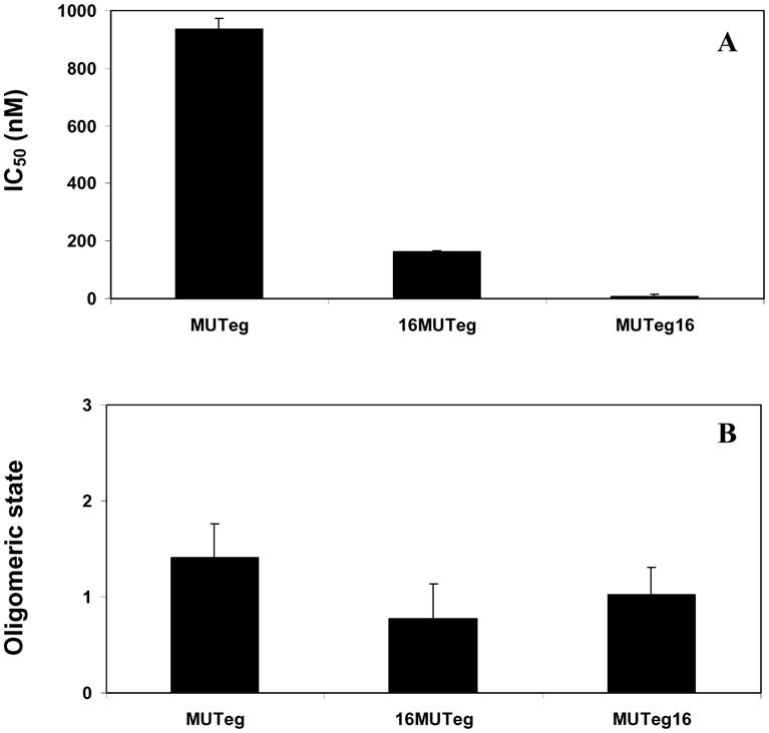
Cell-cell fusion inhibition assay for the N36 mutants as well as their fatty acid conjugates. (A) Fusion inhibition induced by the N36 MUTe,g peptides. The *IC_50_* values of the different peptides are presented. For each peptide at least four independent experiments were performed and were included in the calculation of the standard deviation. (B) The inhibitory oligomeric state of the peptides. The Hill's coefficient parameter for the different peptides is presented. For each peptide at least four independent experiments were performed and were included in the calculation of the standard deviation.

In order to investigate further the mechanism of inhibition we utilized known N36 mutants [20] (see Table 3 for sequences). The first was N36 MUT*e*,*g* which contains mutations in its *e* and *g* positions. These mutations preserve its ability to self-assemble into trimers, but it cannot interact with the CHR. The second mutant was N36 MUT*a*,*d* which contains mutations in it's *a* and *d* positions knocking out its ability to interact with itself, thus leading to inability to create the internal coiled-coil. These mutants demonstrated that the NHR can inhibit by preventing the formation of the viral NHR coil-coiled (probably as a monomer or dimer), or by binding to the CHR domain to prevent SHB formation (probably as a trimer). We conjugated a palmitic acid to the N or C- terminus of both of them (Table 3) and determined their IC_50_ inhibitory values. As expected, N36 MUT*a*,*d* was inactive alone and when conjugated to palmitic acid, because it could not bind itself, as well as CHR, therefore both modes of inhibitions could not take place. Strikingly however, the attachment of palmitic acid to N36 MUT*e*,*g* caused an increase of 7-fold to 100-fold in its IC_50_ compared to the soluble peptide, depending on the directionality of the conjugation. N36 MUT*e*,*g*, C16-N36 MUT*e*,*g*, and N36 MUT*e*,*g*-C16 exhibited *IC_50_* values of 936±36, 162±4, and 8.8±4 nM, respectively (Figure 5). Such preference was not observed with the wild type N36 which preserve binding to the CHR region. The data analysis suggested a trimeric and monomeric modes of inhibition for the wild type N36 and its palmitic acid conjugates, respectively (Figure 2B). Here, N36 MUT*e*,*g*, C16-N36 MUT*e*,*g*, and N36 MUT*e*,*g* present oligomerization parameters values of 1.4, 0.77, and 1.4 respectively (Figure 5B), suggesting primarily a monomeric mode of inhibition. The *c* parameter of N36 MUT*e*,*g*, C16- N36 MUT*e*,*g*, and N36 MUT*e*,*g*-C16 was compared to the *c* parameters of N36, N36M, C16-N36, N36M-C16, and to themselves by the nonparametric Mann-Whitney test. Even though our sample size is small, out of 14 comparisons only one did not obey our predictions.

**Table 3:** Sequences, designations, and IC_50_ values of the N36 mutated peptides and their lipophilic conjugates.

Designation	X	Peptide sequence	Z	IC_50_(nM)
N36 MUT*e*,*g*	H	**X**- SGIDQEQNNLTRLIEAQIHELQLTQWKIKQLLARILK		936±36
C16-N36 MUT*e*,*g*	C16-	**X**- SGIDQEQNNLTRLIEAQIHELQLTQWKIKQLLARILK		162±4
N36 MUT*e*,*g*-C16	H	**X**- SGIDQEQNNLTRLIEAQIHELQLTQWKIKQLLARILK(δ-NH**Z**)	-C16	8.8±4
N36 MUT*a*,*d*	H	**X**- SGIVQQLNNQLRAEEANQHLEQLSVWGSKQNQARRLK		inactive
C16-N36 MUT*a*,*d*	C16-	**X**- SGIVQQLNNQLRAEEANQHLEQLSVWGSKQNQARRLK		inactive
N36 MUT*a*,*d*-C16	H	**X**- SGIVQQLNNQLRAEEANQHLEQLSVWGSKQNQARRLK(δ-NH**Z**)	-C16	inactive

### Comparing Fusion Inhibition Results to Those Obtained with a Reporter Gene Assay

Our cell-cell fusion assay is based on lipophilic fluorescent probes. Therefore, there is a risk that the inhibitory results that we obtained are due to hemifusion. In order to exclude this possibility, we performed a reporter gene cell-cell fusion assay for representative peptides as a proof of concept. The gene reporter assay is based on activation of HIV long terminal repeat-driven luciferase cassette in TZM-bl (target) cells by HIV-1 tat from the HL2/3 (effector) cells. The peptides that we examined were: N36, N36M, N36M-C16, and N36 MUT*e*,*g*-C16. Their original IC_50_ values (nM) were: 488, 531, 159, and 8.8 respectively, in comparison to: 472, 333, 128, and 8 respectively, in the gene reporter assay experiment. Since the values were comparable we conclude that the inhibitory results obtained with our cell-cell fusion assay represent full fusion and not hemifusion.

### The Relative Binding of Labeled Peptides to the Membrane of Specific Cell Populations

**Figure 6:**
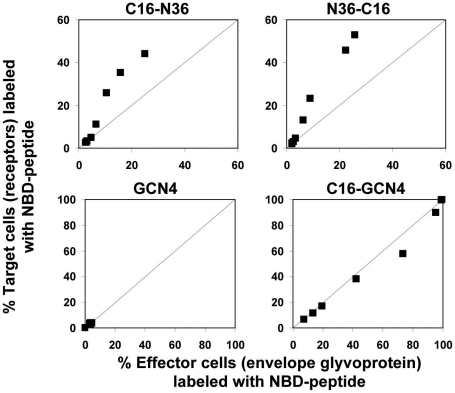
Relative binding of labeled peptides to the membrane of specific cell populations. In each panel the Y axis represents the percentage of target cells (with receptors) labeled with NBD-peptide whereas the X axis represents the percentage of effector cells (with envelope glycoprotein) labeled with NBD-peptide. The lower panels illustrate two controls utilized: _NBD_GCN4 as a non-binding peptide and C16-_NBD_GCN4 as a strongly non-specific binding peptide. A line is drawn in each panel to emphasize where we would expect the data in case there is no preference between the different populations. The different data points represent rising peptide concentrations.

To examine whether the peptides have an enhanced tendency to bind the cells with the receptors (target cells), or those with the ENV glycoprotein (effector cells), in a dynamic fusion process, we employed a triple staining assay. Fluorescently labeled peptides were incubated with differently labeled effector and target cells, exactly according to the protocol of the cell-cell fusion assay. The fusion was allowed to take place and then the sample was washed and measured by FACS. Further analysis, as specified in the Materials and Methods section, enabled us to compare the relative level of the peptide's binding for each cell population (Figure 6). The _NBD_GCN4 peptide served as a negative control for a non-binding peptide, whereas C16-_NBD_GCN4 served as a positive control for a strongly binding peptide without preference for a specific cell population. A line is drawn in each panel to emphasize where we would expect the data in case there is no preference among the different populations. Since the _NBD_GCN4 peptide does not bind the membranes, all the data points are concentrated in the lower left-hand corner. We can conclude that (in the same conditions as for the experiments determining the inhibitory activity of the peptides) there is a tendency of the conjugated N36 peptides to reside more on target than on effector cells.

## Discussion

**Figure 7:**
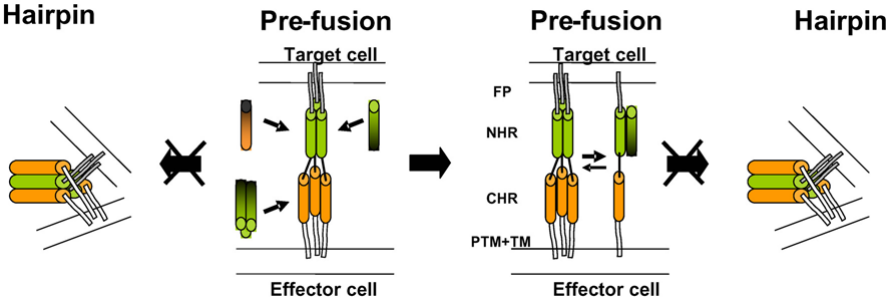
A cartoon illustrating possible modes of inhibition. The NHR region is denoted by green cylinders, the CHR region is denoted by orange cylinders, and the fusion peptide is denoted by a black line. The C-terminus of the inhibitory N- or C-peptides is represented by a black color. The Pre-fusion conformation is presented on the left of the middle panel. N36 can bind the Pre-fusion conformation in two ways: It can bind the CHR region and inhibit progression into the Post-fusion conformation, or it can interrupt the creation of the central NHR coiled-coil by driving the equilibrium towards the dimeric and monomeric forms (here only the monomeric form is presented for simplicity) thereby preventing progression into the Post-fusion conformation. CHR can only bind the NHR region in the Pre-fusion conformation thereby preventing fusion.

Using synthetic peptides with homologous sequences to endogenous domains within gp41 is a powerful tool to decipher the molecular mechanism of HIV-cell fusion. Among these peptides the NHR and CHR play a crucial role. Studies with soluble CHR derived peptides support the current model in which gp41 adopts an extended conformation in the pre-fusion step, inserts the fusion peptide into the target membrane while the NHR forms a trimeric coil-coiled structure. A critical step toward membrane fusion is the collapse of this structure to form the SHB. CHR-derived synthetic peptides can prevent SHB formation by competing with the endogenous CHR domain for the binding of the NHR trimers (Figure 7). For such an inhibition to occur, CHR needs to bind in an antiparallel manner to the NHR. To support this mechanism, we have previously anchored short CHR peptides to the cell membrane by palmitic acid conjugation. The CHR derived lipopeptides had 30-fold higher inhibitory activity when attached via their C-terminus (antiparallel to the endogenous NHR), compared to the N-terminus. That study also demonstrated the C-terminal boundary of the six helix bundle [22].

### The Mode of Inhibition of NHR

**Figure 8:**
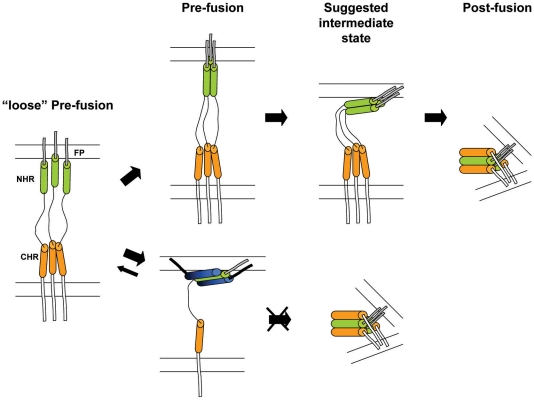
A cartoon illustrating a plausible model for the fusion process and its inhibition by the long fatty acid conjugated peptides. The NHR region is denoted by green cylinders, the CHR region is denoted by orange cylinders, the inhibitory N36 peptide is denoted by blue cylinders (blue and black for the N- and C-terminal of the peptide, respectively), and the fatty acid is denoted by a thick black line. On the left is the conformation immediately after the binding of the receptors and co-receptors, in which the NHR coiled-coil has not been created yet- “loose” pre-fusion. If the NHR region, as well as the N36 peptide, has a planar orientation towards the cell membrane, it can explain the directionality independence of fatty acid conjugation. Here, we show only the interference with the formation of the trimeric coiled-coil since it appears to be the main inhibitory mode of the conjugated N36 peptides.

NHR peptides display a distinct feature in comparison to the CHR peptides in their ability to self oligomerize in solution [34],[35]. Thus, they can bind to two endogenous domains of gp41 in the pre-fusion extended conformation [20]. Synthetic NHR can bind the endogenous NHR to prevent the formation of the coil-coiled NHR trimer probably as dimers or as monomers (Figure 7). In addition, NHR can bind the CHR and hence prevent endogenous SHB core formation (Figure 7). Binding to the CHR region depends on the ability of the NHR to homo-oligomerize [20], probably as a trimer. In support of this, enhancing the trimeric tendency of N36 increases its inhibitory activity [36],[37]. The accepted model is that this trimeric N36 form is primarily responsible for binding of the CHR region. In both of these mechanisms the directionality of the NHR towards the internal pre-fusion conformation seems to be important. To test this hypothesis we conjugated fatty acids with increasing lengths to the N or C-terminus of N36, and the inhibitory activity of the resulting lipopetides was examined in a cell-cell fusion assay. Importantly, the IC_50_ of the resulting lipopeptides increased significantly in correlation to the length of the fatty acid (Figure 1), as well as to their ability to bind cells which was examined by a triple staining assay (Figure 3). However, in contrast with the CHR, we found that the directionality of the attachment of N36 was not critical, since the attachment of the same fatty acid to N36 increased its inhibitory activity similarly, independent of whether it was attached to the C- or N-terminus (Figure 1). These findings suggest a planary orientation of the endogenous NHR region, as well as the N36 lipopeptides, on the cell membrane. Indeed, previous studies have revealed that NHR derived peptides can bind and assemble on a membrane and adopt an α-helical structure [38],[39],[40],[41],[42],[43]. Since it is less likely that internal coiled-coil will disassemble after its creation, we suggest that a loose extended conformation is created after the conformational changes induced by the receptors and co-receptors binding. In this conformation the FP is inserted into the host cell membrane but the internal coiled-coil is not formed yet (“loose” Pre-fusion in Figure 8). Then, the NHR coiled-coil is formed which leads to its parallel orientation towards the membrane, and finally folding into the post-fusion conformation. In this model the peptides with the long fatty acid will create a chimeric coiled-coil with endogenous NHR leading to an altered sequence of events as is presented in Figure 8 at the bottom. However, it is possible that the conjugated peptides inhibit partially also from solution. We suggest that these gp41 conformations: the loose pre-fusion conformation and the NHR region lying parallel to the cell membrane, are additional intermediate conformations during the fusion process.

We observed two different shapes of the inhibition curves of N36 and its fatty acid derivatives: sigmoid for the N36 and short fatty acids conjugated peptides, in contrary to hyperbolic for the longer fatty acid conjugates (see Figure 2A). We utilized a derivative of Hills' equation for the fitting of the experimental cell-cell fusion assay data, and for extracting the *IC_50_* value, in which the Hill coefficient represents the oligomeric tendency of the peptide in the inhibition process. Examining the oligomeric parameters revealed an interesting trend. The soluble unmodified N36 and N36M peptides act as trimers, whereas the strongly membrane bound lipopeptides C16-N36 and N36M-C16 act as monomers (see Figure 2B). We speculated that N36 mostly binds the CHR as a trimer (However, the monomeric fraction of these peptides can also bind the endogenous NHR) while C16-N36 mainly binds the endogenous NHR as monomers.

To further support this, we conjugated palmitic acid to the N- or C-terminus of two previously studied N36 mutants: (i) N36 MUT*e*,*g* which contains mutations in its *e* and *g* positions resulting in its inability to interact with the CHR, and (ii) N36 MUT*a*,*d* which contains mutations in it's *a* and *d* positions knocking out its ability to interact with itself, leading to inability to create the internal coiled-coil [20]. Fatty acid conjugation to N36 MUT*a*,*d* could not compensate for the inhibitory obligatory requirement of N36 self binding. In contrast, fatty acid conjugation dramatically increased the inhibitory activity of N36 MUT*e*,*g* (up to 100-fold). In this case the C-terminal anchored N36 MUT*e*,*g*-C-16 is about 20-fold more active than the N-terminal anchored C-16-N36 MUT*e*,*g*. Importantly, N36 MUT*e*,*g*, C16-N36 MUT*e*,*g*, and N36 MUT*e*,*g* can bind the NHR but not the CHR. The fitting of their inhibitory curves reveal that they bind the endogenous NHR region in a monomeric form (Figure 5B).

The enhanced inhibitory activity of the conjugated peptides can be accounted for by: (i) increased local concentration of the conjugated peptides on the membrane surface resulting in increased accessibility near the fusion site. (ii) If conjugation of a fatty acid indeed changes the tendency of the peptide into a monomeric inhibitory mode of action, then less peptides are required to exert the same inhibitory effect thus reducing the *IC_50_* value, and (iii) When N36 inhibits it can bind simultaneously to the NHR and CHR regions of the same pre-fusion structure. In contrast, when a peptide can only bind one target site (like N36 MUT*e*,*g* or the monomeric conjugated peptides) a lower concentration exerts the same effect thus reducing the *IC_50_* value. Interestingly, analysis of the inhibitory curves of C-helix peptides also reveal, as expected, a monomeric mode of action (data not shown). Since, similarly to the anchored N36 MUT*e*,*g*, a C-helix peptide can bind only the NHR, the inhibitory activities of N36 MUT*e*,*g*-C16 and e.g. T-20 are similar and in the low nanomolar range.

Combining these results it seems that N36, N36M or the lipopeptides with the short fatty acids primarily target the endogenous CHR region as trimers, and that conjugation of a long fatty acid leads to a shift toward a lower oligomerization requirement for the inhibition reaction, thereby primarily targeting the endogenous NHR region. Apparently, the membrane bound peptide does not depend on trimerization for the inhibition activity similarly to the N36 peptide in solution; membrane binding compensates for the trimerization requirement. Strengthening this assumption is another interesting finding - a tendency of the N36 conjugates to reside more on the target cells that occupy the receptors than on the effector cells (Figure 5). This feature was detected by a triple staining assay performed under the same protocol conditions utilized for the cell-cell fusion assay.

A new anti-HIV-1 therapeutic category classified as fusion inhibitors emerged to the HAART (Highly Active Antiretroviral Therapy) with the entry of a C-peptide named enfuvirtide. The potential of C- and N-peptides to inhibit the fusion process of the virus was discovered simultaneously. Nevertheless, most efforts were aimed at developing C-peptides as drugs. This was due to inferior inhibitory activities demonstrated by N-peptides, which were attributed to their tendency to form weakly active oligomers. Studies that have demonstrated improved inhibition of N-peptides are rare and include (i) Stabilization of a specific, usually a trimeric, coiled-coil NHR complex, by fusion to unrelated coiled-coils [36], by covalently connecting NHR regions [37],[44],[45], or by combining different methods including point mutations in specific heptad repeat positions [17]. (ii) Creation of an incomplete core complex [46]. (iii) Abolishing the CHR binding capability by altering the *e* and *g* positions of the N36 heptad repeat [20], resulting in enhanced inhibition, probably due to reduced aggregation. Most of these methods involve elaborate techniques. Here we have demonstrated a significantly enhanced inhibitory activity of an N-peptide by a simple chemical reaction that involves the attachment of a fatty acid to N36. The similarity between functional regions in the envelopes of many viruses suggests a possible new therapeutic approach.

In summary, taking all of the results into consideration leads us to suggest that our peptides demonstrate a shift in the inhibitory mode of action from mainly a trimeric, oligomeric N36/N36M complex, which can target either the internal NHR coiled-coil or the CHR region, to monomeric lipopetides that mostly target the internal NHR coiled-coil. Additionally, the similar inhibitory effect of the N- and C-terminally conjugated peptides suggests that the mode of inhibition involves a planary peptide orientation on the membrane's surface indicating a possible additional intermediate conformation during the fusion process. (Figure 8). Importantly, this study demonstrates that a simple chemical conjugation of fatty acids to N36 can significantly increase its inhibitory activity.
